# Could IGF-I levels play a neuroprotective role in patients with large vestibular schwannomas?

**DOI:** 10.4155/fsoa-2017-0103

**Published:** 2017-11-10

**Authors:** George Fotakopoulos, Kostas Fountas, Eleni Tsianaka, Polikceni Kotlia, Dimitrios Pachatouridis, Thanos Paschalis, Spyridon Voulgaris

**Affiliations:** 1 Department of Neurosurgery, University Hospital of Thessaly, University Hospital of Larissa, Biopolis, 41110 Larissa, Thessaly, Greece; 2 Department of Head of Critical Care, University of Thessaly, University Hospital of Larissa, Biopolis, 41110 Larissa, Thessaly, Greece; 3 Department of Neurosurgery, University Hospital of Ioannina, Ioannina, Greece

**Keywords:** IGF-I, neuroprotection, vestibular schwannoma

## Abstract

**Aim:**

To evaluate the possible superiority of outcome in patients with elevated IGF-I levels after vestibular schwannoma (VS) resection.

**Patients & methods:**

This retrospective study included 65 patients (34 male, 52.3%) with VS operated in between January 2009 and April 2014 (follow-up 3.2 ± 0.7 years). Preoperative or postoperative IGF-I levels were identified for each patient.

**Results:**

Patients were divided into two groups: Group A (small size tumor), 56 patients; and Group B (large size tumor), 9 cases. IGF-I levels in Group A (195.8 ± 32.9 ng/ml) were compared with those of Group B (242.2 ± 22.2 ng/ml) and were found to have statistically significant difference (p = 0.001).

**Conclusion:**

Increased IGF-I levels could hold a key role in nerve recovery in patients undergoing surgical resection of large VS.

## Lay abstract

The aim of this study was to examine the possible correlation between IGF-I levels in serum in large vestibular schwannomas (VS) and its potential neuroprotective effect on the vestibulocochlear nerve. This retrospective study included 65 patients that underwent surgery, 9 of whom were diagnosed with large VS. Results suggested that elevated IGF-I in serum levels could help identify patients with large VS with better neuroprotective activity.

Vestibular schwannoma (VS) occurs in 6% of all intracranial tumors and in 80% of cerebellopontine angle (CPA) tumors [1]. The incidence in many countries has increased over the last 20 years from 7.8 to 17.4 cases per million population per year [1]. Most cases present with unilateral VS in their 40s and 50s [1] with symptoms arising from the 8th cranial nerve (CN) compression (sensorineural hearing impairment [SNHI; 72–90%] [2–4] and tinnitus [70%]) [5,6]. There are also patients who present with symptoms from the 5th CN (facial numbness) and brain stem compression as well as gait ataxia, symptoms from the 3rd CN, including headache, nausea, diplopia, hoarseness, with dysphagia from 9th–10th–12th CNs and obstructive hydrocephalus [1].

According to the Koos grading system, there are four grades as far as the size is concerned [7]. Grade 1 is a small tumor, purely intracanalicular, extending into the CPA without being in contact with the brain stem. Grade 2A and B corresponds to small tumors: for Grade A, the tumor does not extend more than 10 mm into CPA, and for Grade B, the tumor extends 11–18 mm beyond porus acousticus. Grade 3 refers to a moderately large tumor (20–30 mm) occupying the CPA without contact with the brain stem [1]. Grade 4 defines a large tumor (>30 mm) with brain stem and CN displacement [7]. On the other hand, growth rate in the first 2 years, existence of cystic part, hemorrhagic features and hormonal therapy identify a strong growth and are predicting factors for those tumors [8].

Somatomedin C or IGF-I, is an insulin analog, protein macromolecule (hormone). The potential neuroprotective role of IGF-I has been confirmed in many studies [9,10]. IGF-I was found to prevent the loss of choline acetyltransferase activity in embryonic spinal cord cultures as well as to reduce the programmed cell death of motor neurons *in vivo* during normal development or following axotomy or spinal transaction [9]. Also, IGF-I accelerates recovery from sciatic nerve crush in mice, and this results in elevated serum levels of IGF-I that are similar to those obtained from following subcutaneous injections of formulated recombinant human IGF-I [9]. In amyotrophic lateral sclerosis, IGF-I production augments the production of glial-derived neurotrophic factor and accelerates neurite outgrowth without adversely affecting human spinal stem cell proliferation or terminal differentiation [10].

The evidence that IGF-I stimulates myelin expression [11] is probably the underlying mechanism for the stimulating effects of IGF-I on remyelination [12,13]. VS is the most frequent of the merlin-deficient central nervous system tumors [14]. Merlin belongs to the Ezrin–Radixin–Moesin protein family and regulates growth factor receptor signaling [15,16]. The IGF-I receptor is strongly overexpressed and activated in human primary schwannoma cells. IGF-I and IGF-II are overexpressed and released from schwannoma cells. IGF-I stimulates myelination in Schwann cells, which are all strongly activated in VSs [17]. However, this observation is not consistent with findings from many studies that observed direct association between serum IGF-I and the risks of VSs [18].

IGF-I has potent neurotrophic and neuroprotective effects, and extensive preclinical evidence supports the hypothesis of attenuation of motor neuron loss and maintenance of neuronal synapses and neuromuscular junctions by IGF-I [19]. Thus, the aim of this study was to examine the possible correlation between IGF-I serum levels mainly in large VS and its potential neuroprotective effect on the vestibulocochlear nerve, as this may be helpful in its management and surgical plan.

## Patients & methods

This was a retrospective study, affecting VS cases that underwent surgery with a retrosigmoid suboccipital approach, between January 2009 and April 2014 (64 months duration). The follow-up was 3.2 ± 0.7 years (from 12 to 84 months). The final admission to the study was decided after histopathology revealed the presence of typical VS tumor. All patients were preoperatively assessed by neurological examination, computed tomography and MRI. Preoperative or postoperative after histopathological diagnosis, IGF-I levels were identified for each patient. In the European Prospective Investigation into Cancer and Nutrition (EPIC), body height was associated with IGF-I levels in men but not in women [18]. Acromegaly, a condition resulting from excessive growth hormone secretion, has also been found to be associated with an increased risk of Central Nervous System (CNS) tumors [18]. Thus, in this study, we excluded males with body height >190 m, as well as patients with acromegaly.

Transcranial motor evoked potentials (TcMEP) and continuous monitoring of the facial nerve were available intraoperatively via an experienced neurophysiologist. First, the tumor portion just outside the porus acusticus was removed, in order to avoid facial nerve injury, followed by removal of the intrameatal portion (if there was one). Then the residual tumor was removed by sharp dissection from the facial nerve bidirectionally in a piecemeal fashion. In all total resection cases, the capsule was completely removed. Postoperatively all patients had regular follow-up examinations.

### Identification of IGF-I levels

Serum IGF-I was measured with Mediagnost's ELISA kit (Reutlingen, Germany). From each subject, 5 ml of blood were collected into a separator gel tube for serum isolation, which was subsequently divided into aliquots and stored in a frozen matter at -20°C for later analysis. The expected normal values for adults after puberty are 150–350 ng/ml. Samples were taken from 10.00 am to 11.00 am, in order to avoid the wide ranging values of serum of IGF-I during the day.

### Statistical analysis

Data are presented mean ± SD. Data were assessed for normality using the Shapiro–Wilkes test. Nominal data were analyzed using the Fisher's exact test. Continuous data were analyzed using the Student's t-test or the Mann–Whitney U-test as appropriate. A p-value <0.05 was considered as statistically significant. Statistical analyses were performed with the use of Statistical Product and Service Solutions (SPSS) software, version 15 (SPSS, Inc., IL, USA).

Receiver operating characteristic (ROC) analysis was performed to demonstrate the neuroprotective effect of IGF-I levels in the outcome of patients who underwent surgery for large VS. A p-value <0.05 was considered as significant. Statistical Package for the Social Sciences (SPSS.11; IL, USA) was used for analysis.

## Results

In this study, a total of 65 patients (34 male, 52.3%) underwent surgery with retrosigmoid suboccipital approach for VS. They were divided into two groups: the first group (Group A) included 56 patients (30 male, 53.5%), mean age (64.3 ± 6.1) years (range 27, 47–74 years) with small size tumor, and the second group (Group B) involved 9 cases (4 male, 44,4%), mean age (64.6 ± 6.3) years (range 16, 58–74 years) with large size tumor (≥2.5 cm) (Table 1). The IGF-I levels in Group A were 195.8 ± 32.9 ng/ml, and when compared with those of Group B (242.2 ± 22.2 ng/ml), these were founded to show statistically significant difference (p = 0.001). As it was also shown in Table 1, the obstructive hydrocephalus present on the day of admission, nausea, diplopia and hoarseness and/or dysphagia were found to have a statistically significant correlation between the two groups (p < 0.05).

**Table 1.** **Baseline characteristics of patients.**

**Name**	**Group A (small size) n = 54**	**Group B (large size) n = 9**	**p-value**
Age (years)	64.3 ± 6.1	64.6 ± 6.3	0.894

Sex (male), n (%)	30 (53.5)	4 (44.4)	0.726

IGF-I (ng/ml)	195.8 ± 32.9	242.2 ± 22.2	0.001

Total resection	54 (96.4)	8 (88.8)	0.365

Obstructive hydrocephalus in day of admission, n (%)	0 (0)	6 (66.6)	<0.005

**Symptoms from the 5th CN compression**			

Headache, n (%)	30 (53.5)	9 (100)	0.009

Nausea, n (%)	16 (28.5)	9 (100)	0.005

Diplopia, n (%)	2 (3.5)	4 (44.4)	0.002

**Symptoms from the 9th–10th–12th CNs compression**			

Hoarseness and/or dysphagia, n (%)	0 (0)	4 (44.4)	0.005

Data are presented as mean ± SD, otherwise is indicated.

CN: Cranial nerve.

### Outcomes

Clinical outcomes are shown in Table 2. The incidence of SNHI in Group B was 4/9 cases (44.4%) with no statistical significance between two groups (p = 0.038). Facial nerve palsy also was without statistical difference (p = 0.135). In addition, the intensive care unit stay and the length of hospital stay were statistically significant with p < 0.05.

**Table 2.** **Patients outcomes.**

**Name**	**Group A (small size) n = 56 (%)**	**Group B (large size) n = 9 (%)**	**p-value**
Sensorineural hearing impairment, n (%)	7 (12.5)	4 (44.4)	0.038

Facial nerve palsy, n (%)	7 (12.5)	3 (33.3)	0.135

ICU stay (days)	3.6 ± 1.0	11.1 ± 4.3	<0.005

Length of hospital stay (days)	12.2 ± 2.2	24.4 ± 6.3	<0.005

Data are presented as mean ± SD, otherwise is indicated.

ICU: Intensive care unit.

**Figure 1.**
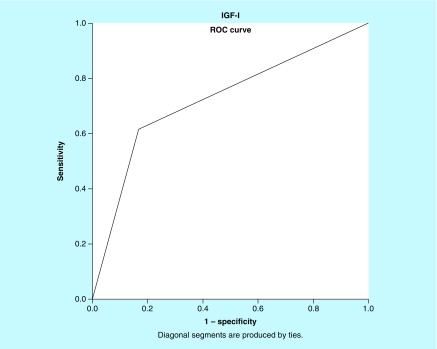
**ROC analysis: Receiver operator characteristic analysis showed that IGF-I presented the best performance for predicting favorable outcome among other variables for patients that underwent surgery for large VS with an AUC (standard error) of 0.27 (0.06), p = 0.05; an IGF-I value of >220 ng/ml presented with 70% sensitivity and 78% specificity.** VS: Vestibular schwannoma.

Notably, ROC analysis showed that IGF-I presented the best performance among other variables assessed with an AUC (standard error) of 0.27 (0.06), p = 0.05; an IGF-I value of >220 ng/ml presented with 70% sensitivity and 78% specificity (Table 3 & Figure 1).

**Table 3.** **Performance of variables for the prediction of the outcome for patients that underwent surgery for vestibular schwannoma after receiver operating characteristic (ROC) analysis.**

**Parameters**	**IGF-I**	**Total resection**	**Hydrocephalus**	**ICU stay**	**Hospital stay**	**Headache**	**Nausea**	**Diplopia**	**Hoarseness and/or dysphagia**
AUC	0.275	0.503	0.614	0.643	0.599	0.500	0.583	0.614	0.564

Standard error	0.068	0.078	0.081	0.079	0.083	0.078	0.078	0.081	0.081

p-value	0.005	0.972	0.145	0.068	0.206	1	0.286	0.145	0.414

ICU: Intensive care unit; ROC: Receiver operating characteristic.

Multivariate analysis (Table 4) showed that IGF-I was an independent factor of outcome (SNHI and/or facial nerve palsy [p = 0.01; odds ratio = 0.38; 95% CI = 0.92–1.05]). Only those variables that were significantly associated with favorable outcome in the univariate analysis were entered in the stepwise logistic regression models.

**Table 4.** **Independent risk factors for favorable outcome of patients that underwent surgery for large vestibular schwannoma after multivariate analysis.**

**Name**	**p-value**	**OR**	**95% CI**
IGF-I	0.001	0.383	0.092–1.056

ICU stay (days)	0.008	0.312	0.327–1.597

ICU: Intensive care unit; OR: Odds ratio, P: Value for the difference between groups.

## Discussion

Our findings suggest that an elevated level of IGF-I with value of >220 ng/ml may have a potential neuroprotective effect on vestibulocochlear nerve in patients diagnosed with large VS and thus may be helpful in its management and surgical plan.

The potential neuroprotective effect of IGF-I is based on the finding that IGF-I helps to increase both the maturation and myelinogenic properties of myelin-deficient and normal oligodendrocytes [13].

One of the most common symptoms mainly seen in large VS cases is hearing impairment [2–4]. Uncorrelated results between the IGF-I levels and hearing impairment raise the suspicion that the potential neuroprotective effect of IGF-I is not so efficient or needs enhancing agents. There are studies in other specialty areas that show that the effectiveness of IGF-I is supported by different agents, such as other growth factors in combination [20,21]. It is also possible that the therapeutic – neuroprotective activity of IGF-I to be achieved in higher levels than those pathologically increased in VS cases. In our study, ROC analysis showed that a value of serum IGF-I of >220 ng/ml presented with 70% sensitivity and 78% specificity, and correlated with better clinical outcome (SNHI and facial nerve palsy). Multivariate analysis also showed that IGF-I was an independent factor of SNHI and/or facial nerve palsy (p = 0.001) after large VS surgical excision. This means that the elevated IGF-I serum levels could help patients with large VS and have better neuroprotective activity.

VSs are considered by many surgeons to be one of the most difficult brain tumors to remove without producing dysfunction [22–24]. In large VSs the postoperative facial nerve palsy is over 40% of cases [25]. Complete tumor removal is the procedure of choice, in order to avoid recurrence, but subtotal or near total resection decrease the possibility of facial nerve impairment, such as staged resection [26,27]. Remarkably, in our study, total tumor resection was achieved in 96.4% in Group A and 88.8% of cases in Group B with no statistical correlation between the two groups (p = 0.365).

Tumor size is the main criterion for not attempting a hearing preservation procedure, because the majority of patients with large VSs have already a hearing impairment of some sorts (hearing loss within the 40 dB range) [28]. According to these facts, the goal in the treatment of large VS tumors is mainly to maintain the facial nerve function. At many centers, the ability of neurosurgeons in large VS tumors to preserve facial nerve function is more than 80% with complete tumor removal [25]. In our study, the tumor was completely removed in 88.8% of patients in Group B, and the incidence of facial nerve palsy was 33.3% (3 cases). There are also cases, in the elderly population (>65 years old) for instance, where neither total nor any tumor resection is necessary [29]. Based on these and considering our findings regarding the possible neuroprotective effect of IGF-I, it would be better to perform a subtotal tumor removal in the more elderly population, when the IGF-I levels are elevated >220 ng/ml, in order to avoid any dysfunction. It is known that many patients with an unchanged VS tumor are do not die because of the tumor.

**Figure 2.**
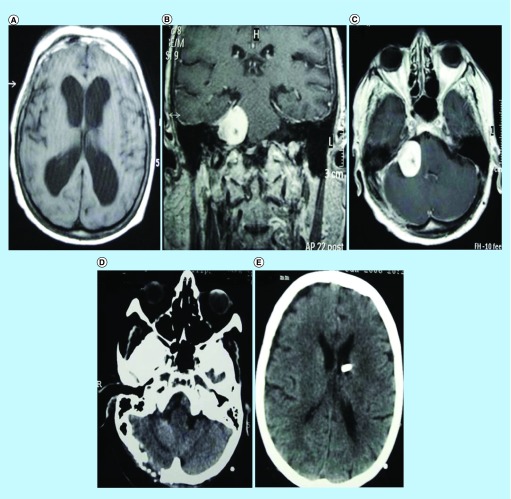
**A Patient imaging illustration: A coronal (B) and axial (C) preoperative MRI to a 73-year-old woman, presenting with obstructive hydrocephalus (A) and hearing loss in the right ear.** The patient underwent a suboccipital retrosigmoid approach with subtotal tumor resection and a operative treatment of the hydrocephalus, viewing at the postoperative CT scans **(D)** & **(E).**

Large VS tumors (>2.5 cm) were associated with a lower risk of development [1] and a tendency toward increasing proliferative activity in patients younger than 50 years [30]. In our study, there was a 73-year-old woman from Group B with subtotal resection and in a follow-up of 3 years, for whom tumor recurrence was not observed (Figure 2). Thus, the criterion for intervention depends on neurological symptoms and patient's age.

Lanman *et al*. report a higher rate (96.3%) of total tumor removal in their series by the translabyrinthine approach, but the incidence of postoperative facial nerve palsy was 47% [31]. The preservation of facial nerve function, after removal of large VS tumor, has been reported to be 60.8–75% [32], and the definition includes both excellent (House-Brackmann [HB] grade ½) and intermediate (HB grade ¾) function [33]. In our data, using the retrosigmoid suboccipital approach, the preservation rate of excellent (HB grade ½) facial nerve function after total resection with continuous intraoperative monitoring (TcMEPs) and the early identification of the root entry/exit zone, was from 67.4% (in Group B: 6 patients) to 87.5% (in Group A: 49 patients) and in both groups was 84.6% (55 patients), showing intermediate (HB grade ¾) function. On the other hand, there have been studies that have reported that the surgical approach did not correlate significantly with the incidence of facial nerve injury (1.58% via the suboccipital and 2.6% via the translabyrinthine approach) [32,34]. Based on those results, our data suggest that the higher rate (67.4–87.5%) in this study versus (60.8–75%) of other reports of facial nerve preservation might be due to the elevated IGF-I levels (>220 ng/ml).

We acknowledge that there are several points of our study that have to be considered when interpreting its results. First, as the histological type of tumor was unclear from the very first moment, and the final inclusion the study was decided after histopathology revealed the presence of typical VS tumor, in a few cases the serum samples were taken during the follow-up. In literature, it is not clear that in which way tumor's resection and recurrence correlates with IGF-I serum levels. In one study, it has been reported that there was no association between IGF-I levels with breast cancer resection and recurrence [35], but other data reported a tendency for higher levels of IGF-I to be associated with distant recurrence in a univariate analysis, although this effect was not significant [36].

Thus, our data are likely to contain several sources of error due to the possible differentiation in values of IGF-I levels compared with its levels into the presurgical period. A new, large, prospective study with a stricter schedule concerning the period where serum samples will be taken, could solve these problems. In addition, it should be pointed out that this was a one center study and the population studied was small.

It is possible that the delivery of agents such as IGF-I could optimize the postsurgical nerve function, through its neuroprotective effect, giving better nerve function recovery. IGF-I is thus a very promising factor in VS cases, and larger studies are needed in order to clarify the role of that agent in VS cases.

## Conclusion

This study showed that there was a correlation between elevated IGF-I levels and postoperative hearing and/or facial nerve impairment in large VS cases. Thus, the association of good surgical plan and equipments (TcMEPs and continuous monitoring), with possible IGF-I-mediated myelin stimulation in injured nerves, could help achieve a better result. It is also possible that increased IGF-I levels hold a key role in nerve recovery. Our study intended to serve as the starting point for others.

## Future perspective

It is becoming clear that a personalized approach to the management of large VS can drastically improve patient outcomes. A variety of different tests needs to be developed to help determine which specific treatments will be beneficial to each unique patient. To this end, IGF-I serum levels and others molecular testing may be proven to be highly effective in identifying which patients with VSs should receive additional therapy.

**Executive summary**

- An elevated levels of IGF-I with value of >220 ng/ml may have a potential neuroprotective effect on vestibulocochlear nerve in patients suffering from large vestibular schwannoma and this may be helpful in its management and surgical plan.
- The highest rate (67.4–87.5%) in this study versus (60.8–75%) of other reports of facial nerve preservation, might was due to elevated IGF-I levels (>220 ng/ml).
